# The use of classification and regression algorithms using the random forests method with presence-only data to model species’ distribution

**DOI:** 10.1016/j.mex.2019.09.035

**Published:** 2019-09-28

**Authors:** Lei Zhang, Falk Huettmann, Xudong Zhang, Shirong Liu, Pengsen Sun, Zhen Yu, Chunrong Mi

**Affiliations:** a Research Institute of Forestry, Chinese Academy of Forestry, Beijing, 100091, China; b Institute of Arctic Biology, Department of Biology & Wildlife, University of Alaska Fairbanks, USA; c Research Institute of Forest Ecology, Environment and Protection, Chinese Academy of Forestry, Beijing, 100091, China; d Department of Ecology, Evolution, and Organismal Biology, Iowa State University of Science and Technology, Ames, IA, 50011, USA; e Institute of Zoology, Chinese Academy of Sciences, Beijing, 100101, China

**Keywords:** Random forests models species distribution, Binary prediction, Numerical prediction, Threshold, Machine learning, Species traits, Climate change, Forestation

## Abstract

Random forests (RF) is a powerful species distribution model (SDM) algorithm. This ensemble model by default can produce categorical and numerical species distribution maps based on its classification tree (CT) and regression tree (RT) algorithms, respectively. The CT algorithm can also produce numerical predictions (class probability). Here, we present a detailed procedure involving the use of the CT and RT algorithms using the RF method with presence-only data to model the distribution of species. CT and RT are used to generate numerical prediction maps, and then numerical predictions are converted to binary predictions through objective threshold-setting methods. We also applied simple methods to deal with collinearity of predictor variables and spatial autocorrelation of species occurrence data. A geographically stratified sampling method was employed for generating pseudo-absences. The detailed procedural framework is meant to be a generic method to be applied to virtually any SDM prediction question using presence-only data.

• How to use RF as a standard method for generic species distributions with presence-only data
• How to choose RF (CT or RT) methods for the distribution modeling of species
• A general and detailed procedure for any SDM prediction question.

## Graphical abstract

**Figure:**
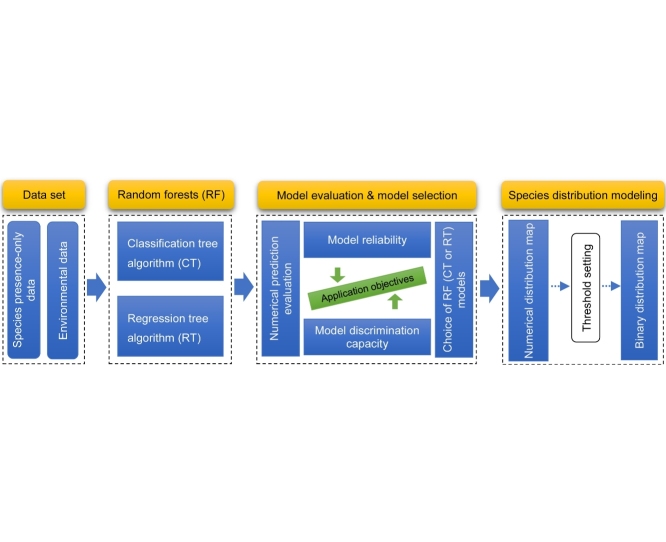

**Specification Table**

**Table:** 

Subject Area:	Agricultural and Biological Sciences Environmental Science
More specific subject area:	Species distribution modelling
Method name:	Random forests models species distribution
Name and reference of original method:	Zhang, L., Huettmann, F., Liu, S., Sun, P., Yu, Z., Zhang, X., Mi, C., 2019. Classification and regression with random forests as a standard method for presence-only data SDMs: A future conservation example using China tree species. Ecological Informatics, 52, 46–56.
Resource availability:	R software

## Method details

**Fig. 1:**
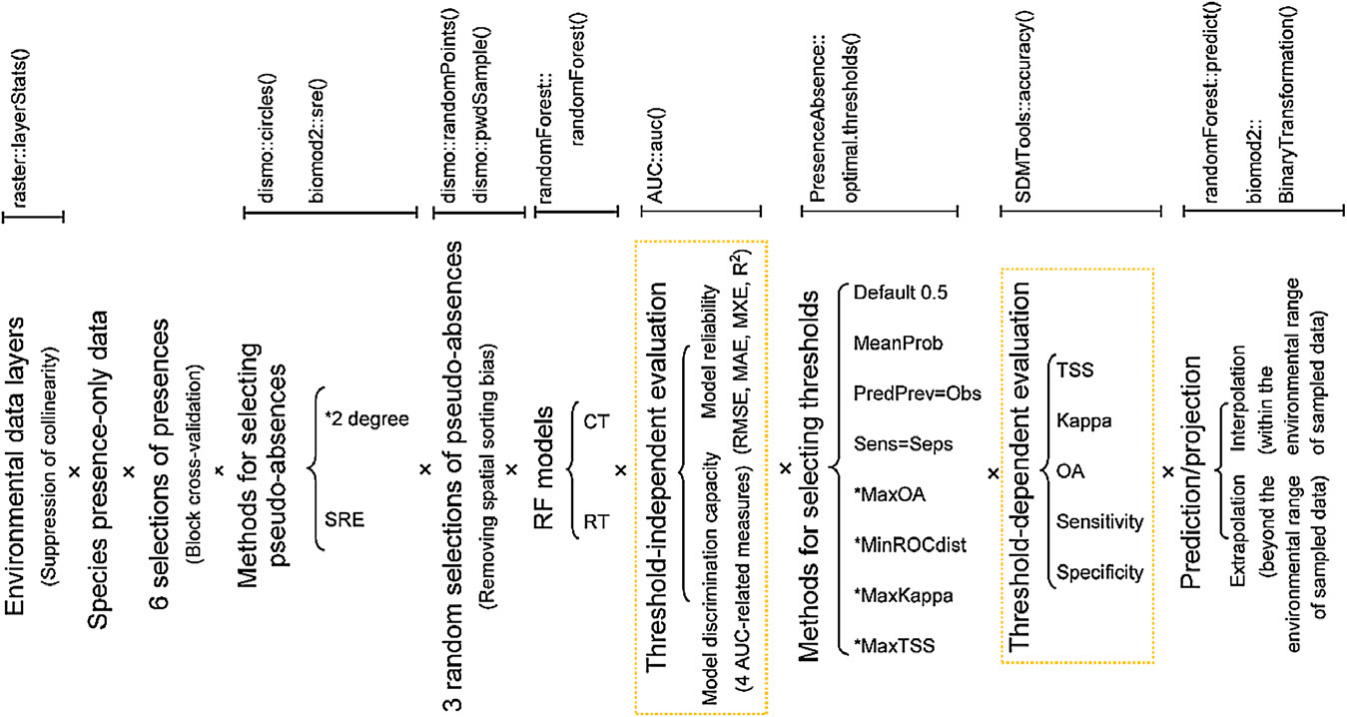
General framework for species distribution modeling by random forests (classification tree (CT) and regression tree (RT) algorithms) and R functions used in this study. Adopted from Zhang et al. [9]; * recommended methods.

Fig. 1 shows the overall workflow using the classification tree (CT) and regression tree (RT) algorithms of the random forests (RF) method to model the distribution of species.

### Data and materials

The study area encompassed all of China. China has a land area of 9.6 million square kilometers and spans a large range of climatic types and natural environments. We characterized the environments in China based on 19 biologically relevant proxy climatic variables (Table 1) drawn from the WorldClim dataset at a resolution of 30 arc seconds (www.worldclim.org). Using the vector map of Chinese administrative regions (http://bzdt.ch.mnr.gov.cn/), the “extract by mask” tool in ArcGIS (ESRI Inc., http://www.esri.com/) was used to delineate climate data within the administrative boundaries of China. Baseline climatic data were obtained from the average of the period 1960–1990, and these data were rasterized to a cell size of 8 km with the “resample” tool in ArcGIS 10.1.

**Table 1:** Biologically climatic variables.

Code	Variable
BIO1	Annual Mean Temperature
BIO2	Mean Diurnal Range (Mean of monthly (max temp–min temp))
BIO3	Isothermality (BIO2/BIO7)
BIO4	Temperature Seasonality
BIO5	Max Temperature of Warmest Month
BIO6	Min Temperature of Coldest Month
BIO7	Temperature Annual Range (BIO5-BIO6)
BIO8	Mean Temperature of Wettest Quarter
BIO9	Mean Temperature of Driest Quarter
BIO10	Mean Temperature of Warmest Quarter
BIO11	Mean Temperature of Coldest Quarter
BIO12	Annual Precipitation
BIO13	Precipitation of Wettest Month
BIO14	Precipitation of Driest Month
BIO15	Precipitation Seasonality (Coefficient of Variation)
BIO16	Precipitation of Wettest Quarter
BIO17	Precipitation of Driest Quarter
BIO18	Precipitation of Warmest Quarter
BIO19	Precipitation of Coldest Quarter

Fifty-two native forest tree species that occur in China were selected for a comparison of the performance of the CT and RT algorithms. The distribution datasets for these 52 tree species were derived from the 1:1,000,000 Vegetation Distribution Map of China [38]. Those data were freely obtained from the Environmental and Ecological Science Data Center for West China of the National Natural Science Foundation of China (http://westdc.westgis.ac.cn). The data were then rasterized to a cell size of 8 km × 8 km with the “polygon to raster” tool in ArcGIS 10.1.

### Suppression of collinearity in predictor variables

Collinearity (or multicollinearity) refers to the strong interdependence of explanatory variables, usually in a regression model. The opposite effect includes two key problems: inflated estimates of a variable’s effect and debated model extrapolation. There are currently many methods available for tackling collinearity problems [1]. *A priori* variable selection (leaving out the most correlated variables) and combining correlated variables into new explanatory terms (e.g., via PCA) are the most often used approaches.

We applied a pre-selecting variables approach to avoid the risk of multicollinearity. We eliminated the predictor variables yielding correlation values above 0.8 (Pearson’s coefficient) in the pairwise cross-correlation matrix, and the remaining variables were used for constructing CT or RT models. We kept the following five climatic variables for the CT and RT models: annual mean temperature, annual temperature range, isothermality, annual precipitation, and precipitation seasonality (coefficient of variation). A correlation matrix was constructed using the “layerStats” function in the “raster” R package [2].

The correlation values chosen above (e.g., 0.8 and 0.7) are from a folk law without statistical foundation, but this choice performed nearly equally well with other approaches addressing collinearity [1]. We proposed the pre-selecting variables approach because of its convenience when used for a large number of species. Furthermore, this approach could minimize model overfitting and ensure comparability across model projections. If a specific species is studied, among the highly correlated predictors we can retain the variable that has the highest correlation with species occurrence data. In addition, Pearson correlations (between numeric variables), polyserial correlations (between numeric and ordinal variables), and polychoric correlations (between ordinal variables) can also be calculated if needed.

### Evaluation of model performance

There is now a plethora of metrics for evaluating SDM performance [3,4]. In short, different accuracy measures have different strengths and weaknesses, and none can provide a universal rating for SDM performance. This phenomenon may be ascribed to the fact that different measures have different strategies of weighting the various types of prediction errors (e.g., omission, commission, or confusion), especially for composite metrics that are based on different algorithms and assumptions (e.g., Kappa; overall accuracy, OA). Therefore, we argue for applying multiple performance metrics to evaluate model performance.

#### Threshold-independent evaluation (numerical prediction evaluation)

For numerical prediction, the predictive performances were evaluated using such measures as the root mean square error (RMSE), the mean absolute prediction error (MAE), the coefficient of determination (R^2^), mean cross entropy (MXE), and area under the curves (AUCs) of four threshold-independent measures: the area under the sensitivity curve, the area under the specificity curve, the area under the accuracy curve, and the area under the receiver operating characteristic curve (ROC). The latter four measures related to the AUC were estimated using the “AUC” package in the R statistical environment [5]. Measures of AUC avoid the need to choose a threshold value that separates presence from absence (i.e., it is threshold independent) and in addition describe the overall ability of the model to discriminate between two cases.

The RMSE, MAE, *R*^2^, and the MXE were calculated for the dataset as in Liu et al. [4]:

$$\text{RMSE = }\sqrt{\frac{1}{\text{n}}\sum_{\text{i}\text{=1}}^{\text{n}}{\text{(}p_{i}-o_{i}\text{)}}^{2}}$$

$$\text{M}\text{A}\text{E}\text{ = }\frac{1}{\text{n}}\sum_{\text{i=1}}^{\text{n}}\left|p_{\text{i}}-o_{i}\right|$$

$$R^{2}=1-\frac{1}{n}\sum_{i=1}^{n}{\left(p_{i}-o_{i}\right)}^{2}/\left[p(1-p)\right]$$

$$MXE=-\frac{1}{n}\left[\sum_{o_{i}=1}\text{ln}p_{i}+\sum_{o_{i}=0}\text{ln}{(1-p}_{i})\right],$$

where *p_i_* and *o_i_* are the predicted and observed values (1 for presence, and 0 for pseudo-absence) for site *i*, $\bar{o}$ is the mean of the observed values, *n* is the total number of sites, and *p* is the observed prevalence of model-testing data.

For numerical predictions, accuracy measures often characterize two aspects of SDM models: discrimination capacity (e.g., AUC values) and reliability (e.g., RMSE, MAE, and R^2^) [4]. Discrimination capacity measures the ability to discriminate presence and absence based on model predictions. Reliability tells us about how closely predicted probabilities match observed proportions of occurrence, i.e., goodness of fit. The relative importance of reliability and discrimination capacity depends on the use of the model and the experience level of the user [6].

#### Threshold-dependent evaluation (binary prediction evaluation)

The accuracy of binary maps produced by threshold-setting approaches was quantified using measures of accuracy derived from the confusion matrices. These measures included Kappa, the true skill statistic (TSS), OA, sensitivity, and specificity. Kappa, TSS, and OA are composite measures of model performance, as they attribute different weights to the various types of prediction errors (e.g., omission, commission, or confusion). The R package “SDMTools” (“accuracy” function) [7] was used to calculate the values of these metrics.

### Choice of threshold-setting methods (Binary conversion of numerical prediction)

Species distribution models (SDMs) usually produce numerical predictions. However, in conservation and environmental management practice (e.g., reserve design and biodiversity assessment), the information presented as species presence/absence (binary) may be more practical than data presented as probability or suitability. Therefore, a threshold is needed to transform the numerical or suitability data to presence/absence data in conservation and environmental management practice.

The binary conversion process can be conducted using the R package “PresenceAbsence” [8]:

(1) Default 0.5: Taking a fixed default value of 0.5 as the threshold.
(2) MeanProb: Taking the average predicted probability of the threshold-selecting data as the threshold.
(3) PredPrev = Obs: The threshold where the predicted prevalence (the proportion of sites occupied) is equal to the observed prevalence of the threshold-selecting data.
(4) Sens = Seps: The threshold where sensitivity (the proportion of observed presences correctly predicted as presence) equals specificity (the proportion of observed pseudo-absences correctly predicted as pseudo-absence) for the threshold-selecting data.
(5) MaxOA: The threshold that results in the maximum value of overall accuracy (OA) for the threshold-selecting data (see below). OA measures the proportion of correctly classified presences and absences.
(6) MinROCdist: The threshold corresponds to the point on the receiver operating characteristic (ROC) curve (sensitivity against 1-specificity) that minimizes the distance to the top-left corner (0,1) in the ROC plot. The area under the curve (AUC) of the ROC is a threshold-independent model evaluation indicator that is independent of both species prevalence and classification threshold [3].
(7) MaxKappa: The threshold that results in the maximum value of kappa for the threshold-selecting data. Kappa measures the extent to which the agreement between observed and predicted values is higher than that expected by chance alone.
(8) MaxTSS: The threshold that results in the maximum value of the true skill statistic (TSS) for the threshold-selecting data. TSS = sensitivity + specificity −1. TSS has all of the advantages of Kappa but is not sensitive to prevalence [3,37].

When converting numerical predictions into binary predictions, the optimal threshold varies with the choice of threshold-setting method. However, the choice of thresholds has practical consequences for estimating of RF model performance and species range shifts under climate change [9]. Hence, the use of an appropriate threshold appears to be a better choice for binary conversions for RF. Zhang et al. [9] demonstrated that the four threshold methods (MaxKappa, MaxOA, MinROCdist, and MaxTSS) based on the composite model accuracy measures (Kappa, TSS, ROC, OA) are promising objective methods for binary conversions of continuous predictions when presence-only data are available. These four methods can also produce the same threshold using either presence-only data or presence/absence data for CT and RT models. The top four approaches performed equally well in terms of model performance, threshold determination, and range shift projection, and each often performed better than the other approaches. The CT default classification method (default 0.5) was not recommended for binary conversions [9,10].

### Generation of pseudo-absence

SDMs are constructed through a series of methods that relate a set of environmental predictors with species distribution data [11]. Information concerning the distributions of species, frequently from museum and herbarium collections, atlases, plant lists, or from volunteer observation networks, are typically composed of presence-only data. The most effective SDM models most often require data on both species presence and absence in the area [12,13]. RF needs species presence and absence records. One solution is to generate pseudo-absences when no reliable absence data are available and then use these as absence data [14,15]. Thus, presence–absence models are increasingly used when only presence data are available by creating pseudo-absence data [16]. Several recent studies have suggested that pseudo-absence data should be restricted to locations that are documented to be distinctly unsuitable for this species occurrence [14,17]. To improve sampling accuracy, the following method as recommended by Barbet-Massin et al. [16] and Zhang et al. [9] for RF was used to randomly select pseudo-absences.

(1) Geographic distance method: Any points located at least two degrees in latitude or longitude from any presence point were selected as true absences (the 2 degrees method). This method assumes that when closer to a known presence point, it is more likely to find the species. This process can be implemented using the “dismo” package (“circles” function) [18] in the R environment.
(2) Environmentally stratified sampling. The locations where all predictor variables fall within the extreme values (both maximum and minimum limits of each predictor) as determined by species presence sites are defined as areas suitable for the occurrence of a particular species. The remaining locations are termed “potential” absences. This process can be implemented by the surface range envelop model (SRE) in the BIOMOD2 package [19] in the R platform (hereafter, the “SRE” method). However, the SRE method was not proposed for generating pseudo-absences when comparing model performance of CT and RT or for comparing them with other SDMs, since both RF and SRE have a piecewise constant function in nature [9].

### Minimization of spatial autocorrelation in species occurrence data

Spatial autocorrelation is the lack of independence between pairs of observations at given distances in space. This is a common phenomenon in ecological data. Spatial autocorrelation in ecological data can create Type I errors in statistical analyses and can inflate the significance of measured species–environment relationships in SDMs when non-spatial models are applied. There many methods of dealing with spatial autocorrelation in the field of species distribution modeling [20]. We recommended the block cross-validation strategy [21] to tackle spatial autocorrelation when RF is used as the SDM. The block cross-validation method can increase spatial independence of training and testing datasets and can help to evaluate model transferability rather than just its interpolation accuracy [21]. This matters a lot, because SDMs are often used for projecting species distributions outside the range of environments (in space or time) on which the original model is based. According to the block cross-validation method, the species distribution data area is divided into several geographically non-overlapping areas to split the data into blocks rather than randomly assigning locations to a split.

A large-sample test indicated that four blocks are appropriate when geographically (2 degrees) or environmentally (SER) stratified sampling was used to generate pseudo-absences. This is because a minimum distance at which the autocorrelation in model residuals begins to disappear is about half the geographical range of species occurrence data (data not shown). This method is recommended for modeling the distribution of a large number of species with presence-only data [9]. In this paper, we implemented block cross-validation to divide the presence data area into four geographically non-overlapping areas as follows. Presence records are split into two sets based on their longitude using a meridian as a dividing line. Then, these two halves (with the same longitudinal range) are separately split into two equal parts (with the same latitudinal interval) using parallels.

### Creation of model-training and -testing data and threshold-setting data

According to the block cross-validation method recommended for RF, the presence data area was divided into four geographically non-overlapping areas to split the data into blocks. Each pair of blocks was used in turn as model-training data (model-training presence data, MCp), while the two others were separately used to test the model (model-testing presence data, MVp) and to select the optimal threshold (threshold-setting presence data, TSp).

Using the 2 degrees or SRT method, RF sampled pseudo-absence data from the entire study region. The following procedure was implemented to sample pseudo-absences for compositing complete model-training and -testing data as well as threshold-setting data.

First, we randomly created a sample of 20,000 pseudo-absences (PA1) from the pseudo-absence population that was generated by the 2 degrees or SRE methods. This process was achieved using the “randomPoints” function the R package “dismo” [22].

Second, we created model-training pseudo-absence data (MCpa). The same number of pseudo-absences as a given species’ presences that were used for model-training data (MCp) was randomly selected from PA1.

In the model-building process, we kept the ratio between the number of presences and absences in the calibration and testing dataset constant at 1:1. This is a recommended method used to find the optimal transforming threshold [23] and to achieve the highest model accuracy [16,24,25] when using RF and a presence/pseudo-absence dataset to develop SDMs.

Third, we created model-testing pseudo-absence data (MVpa). The remaining pseudo-absences (PA1−MCpa) from the sample above were used as the “potential” MVpa. The pairwise distance sampling method proposed by Hijmans [18] was used to select final MVpa points for the model-testing set. We conducted this process using the “pwdSample” function in the R package “dismo” [22].

By combining the block cross-validation strategy with the pairwise distance sampling method to select the pseudo-absence points for the model test and threshold selection sets, spatial sorting bias was removed, and thus, the effect of spatial autocorrelation on the performance evaluation was suppressed.

Fourth, we created threshold-setting pseudo-absence data (TSpa). The remaining pseudo-absences (PA1−MCpa − MVpa) were use as the “potential” TSpa. Similarly, the pairwise distance sampling method was utilized to remove spatial sorting bias between threshold-selecting data and model-training data, yielding the final TSpa.

Finally, we created six sets of sub-model-training (MCp + MCpa) data, and each had a set of accompanying model-testing (MVp + MVpa) and threshold-setting data (TSp + TSpa). For each species, these six sets of sub-data constituted a full model-training dataset.

Because chance plays a part in the choice of the pseudo-absences (PA1), we independently repeated this procedure three times. This was done in an effort to reduce variability in the model-building process and subsequent predictions. Thus, 18 (six sub-model-training sets × three full model-training sets) sets of sub-model-training data were created, and each had a set of companion sub-model-testing data and threshold-determining data.

### The use of classification and regression algorithms using random forests to model species distributions

Random forest is an ensemble learning technique. RF by default can yield categorical and numerical species distribution maps based on the classification tree (CT) and regression tree (RT) algorithms, respectively [26]. RF models in the form of CT and RT are commonly and successfully used in species distribution modeling [27]. In statistical terms, CT can also produce probabilistic predictions (class probability) [9]. In RF models, bootstrap samples are applied to construct a large number of decision trees. These trees are then used to predict new data by aggregating the predictions of the trees (i.e., the proportion of votes for classification, or the average for regression; [28]). In a typical CT, the resulting model output is categorical, and the “winning” class for an observation is the one with the maximum ratio of proportion of votes (the default is 1/k, where k is the number of classes). For presence–absence data, the ratio of the proportions of votes for presence or absence ranges from 0 to 1, and the sum of the ratios is equal to 1. As such, the resulting ratio for presence in CT could be taken as a relative index of occurrence (numerical prediction) [39].

Recent gradient theory [29] holds that numerical results convey more information than binary outputs [30]. For mapmaking, we therefore recommend the use of numerical predictions of RT and CT for species distribution modeling.

Many parameters in RF can be manipulated, e.g., the number of trees grown or the number of variables to try at each split. Because the outcome of both CT and RT is not very sensitive to modifications of these parameters [[31], [32], [33]], it was deemed unnecessary to fine-tune all the RF models to their optimal capacity when they were used for a large number of species. Fine-tuning RF parameters may only be necessary when the method is used for a given well-known species.

We constructed six CT (and RT) sub-models using the six sets of sub-model-training data from the same full model-training dataset. These six sub-model predictions constituted full complete predictions, and three replicates of complete predictions were created (see below). CT and RT models were developed using the package “randomForest” [28] in the R environment.

A total of 18 (CT and RT) sub-models were constructed for each species, and each had a set of companion sub-model-testing data and sub-threshold-setting data that were employed to evaluate model performance and to determine the optimal threshold cut-off values, respectively.

**Fig. 2:**
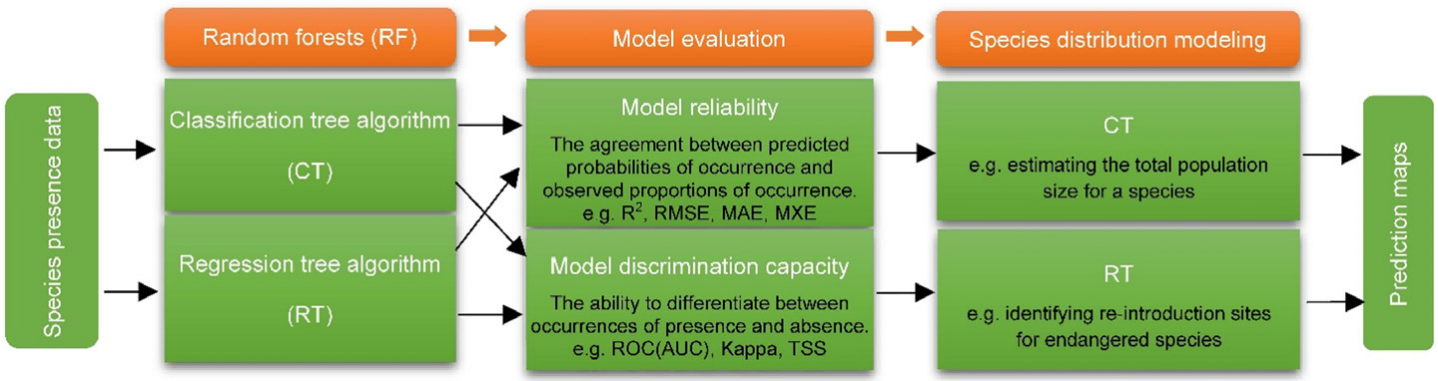
Generic guidelines on how to choose a random forests (classification or regression algorithm) method with presence-only data to model the distribution of species. Adopted from Zhang et al. [9].

**Fig. 3:**
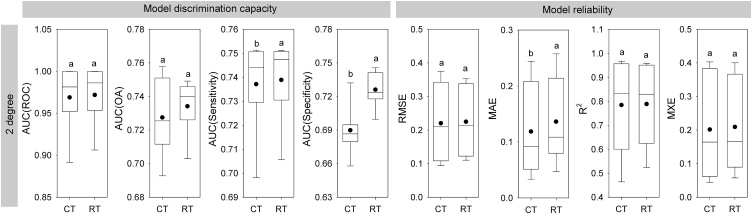
Differences in model accuracy between random forests regression tree (RT) and classification tree (CT) algorithms used for prediction of the distribution of *Quercus serrata*. Dots show the mean value across all species. Different letters indicate significant differences according to a Wilcoxon signed-ranks test (P < 0.05).

We compared the model performance (discrimination capacity and reliability) of CT and RT based on the threshold-independent evaluation metrics. In terms of discrimination capacity, RT performed better than CT, especially for species with restricted ranges; for reliability, CT performed better than RT, especially for species with wide ranges [9]. Therefore, choosing RT rather than CT as the SDM is the best choice if model discrimination capacity is viewed as more important than model reliability, and vice versa (Fig. 2). This can be considered as the generic guideline for choosing RT (CT or RT) algorithms to model the distribution of species, especially for a large number of species. Fig. 3 shows an example for model accuracy of a single tree species.

In practice, the selection between CT and RT depends on the specific species as well as model accuracy measures [4]. Expert experience and ecological common knowledge of the species of interest can at times also be highly effective, albeit nonstandard, evaluation methods. For instance, if SDMs are used to estimate the total population size for a species by predicting the probability of the species occurring at a large number of sites within a region, model reliability should be viewed as more important than model discrimination capacity. When SDMs are used to identify potential re-introduction sites for endangered species, more attention should be paid to model discrimination capacity.

### Ensemble modeling of species distribution

A total of 18 (CT or RT) model predictions were generated for each species. We combined ensemble predictions to draw the final prediction. First, we integrated the numerical predictions generated by the six sub-models within the same one full model set to produce a complete prediction map. The habitat suitability of each site was determined by the maximum predicted values of the six sub-models (assuming their model predictive accuracies were at an acceptable level). Then, we derived the final numerical prediction map for each species by taking the average of the three replicates of complete predictions. Averaging the ensemble predictions is the most often used consensus approach for combining ensemble projections and can significantly improve predictive accuracy [34]. For each species, the averaged prediction resulted in a single prediction at each grid point. According to the above methods, the final numerical prediction maps were generated for each species.

When binary prediction is desired or necessary, the numerical predictions generated by the aforementioned six sub-models within the same one full model set were converted to binary data using the optimal threshold cut-off values corresponding to the six sub-models. We executed this conversion process using the “BinaryTransformation” function in the R package “BIOMOD2” [19]. The sites predicted to be present by at least one of the six sub-models within the same one full model set were considered to be species occurrence sites. Thus, we produced a complete binary map for each full model set. The final binary prediction map consisted of the sites predicted to be present by at least two of the three replicates of complete binary predictions.

A large-sample test showed that four threshold-setting methods (MaxKappa, MaxOA, MinROCdist, and MaxTSS) performed significantly better than other methods and produced the same threshold using either presence-only data or presence/absence data for CT and RT models [9]. Therefore, those methods can be considered as promising threshold methods for RF when only presence data are available. Unless sound justification exists for choosing a particular threshold cut-off over the others (e.g., a good data match); if high sensitivity is needed in defining a management area for a rare species, or if high specificity is needed for determining whether a species is threatened [3,23,35,36], there can be advantages in applying these objective threshold methods for CT and RT. In this manner, map users can choose appropriate threshold cut-off values and generate binary maps according to the intended map use (e.g., species range estimation).

**Fig. 4:**
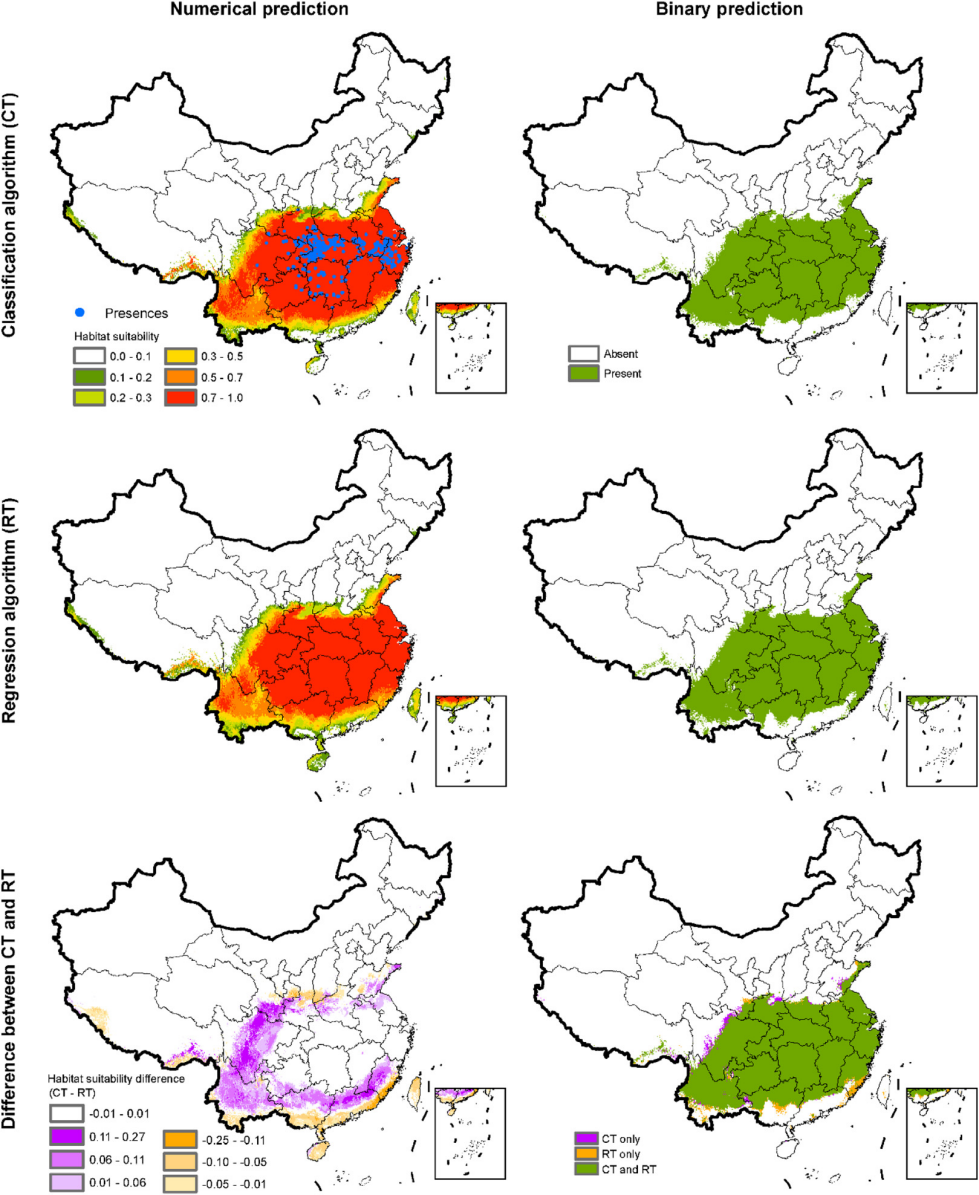
Differences in prediction maps between random forests (RF) regression tree (RT) and classification tree (CT) algorithms for *Quercus serrata*. Numerical predictions were converted to binary predictions through objective threshold-setting methods (MaxTSS).

Fig. 4 demonstrates the spatial difference between CT and RT prediction maps for a single tree species.

### Summary

A detailed procedural framework was proposed for applying RF methods with presence-only data to model the distributions of species (Fig. 1). Choosing RT rather than CT as the SDM is recommended if model discrimination capacity is viewed as more important than model reliability, and vice versa (Fig. 2). MaxKappa, MaxOA, MinROCdist, and MaxTSS are four promising objective methods for binary conversion of continuous predictions when presence-only data are available. Numerical rather than binary prediction distribution maps are recommended, and binary conversion of model outputs should only be implemented when it is clearly justified by the application’s objective. This general procedural framework benefits the wholesale implementation involved with a large number of species because of its simplicity and flexibility.

Due to the complexity of the ecosystem and the uniqueness of the life histories and physiological characteristics of the specific species, these general rules may not apply to all species [10]. Nevertheless, this procedural framework is meant to be a generic concept to be applied to virtually any model prediction question with presence-only data. Under the detailed framework developed in this study, for a specific species, the choice of RF (CT or RT) models can be determined by model performance metrics (model discrimination capacity and reliability). Fine-tuning RF parameters may also be necessary. Other methods that can deal with multicollinearity, autocorrelation, and binary conversions of numerical predictions can be integrated into this procedural framework. Although some SDMs are not very sensitive to collinearity and autocorrelation, they also can embrace these methods.
