# Supplementary material for: The use of classification and regression algorithms using the random forests method with presence-only data to model species’ distribution
Source: MethodsX. 2019 Sep 28;6:2281–92. doi: 10.1016/j.mex.2019.09.035 (PMC6812352; doi:10.1016/j.mex.2019.09.035)
Supplement: Supplementary file 1 [file mmc1.zip › Mendeley Data.zip!/Supplementary figures and tables.docx]

## Appendix A. Detailed description of species traits and their estimation methods.

To investigate which variable best explained the difference in optimal thresholds and in binary predictions among threshold methods, six species traits (specialization, marginality, along with latitudinal, longitudinal, thermal, and elevational ranges) and five threshold-independent model accuracy measures (Kappa, TSS, OA, sensitivity, and specificity) were defined as explanatory variables.

The two measures of environmental niche properties, marginality and specialization, were calculated using the ‘enirg’ function in the R package “ENiRG” (Cánovas *et al.* 2016). Marginality describes the distance of the species optimum from the mean habitat in the study area, and is therefore representative of the species’ ecological niche position. Specialization measures the ratio of the ecological variance in mean habitat to that observed for the focal species. A strong specialization implies that the niche is narrow. The latitudinal, longitudinal, and elevational ranges were described as the differences between the average values of the 10% most extreme sites (maximum and minimum) where a species was present, respectively. The principal component analysis (PCA) was performed on the three thermal variables (annual mean temperature, isothermality, and annual range of temperature) to determine the species thermal range. The first two axes of this PCA, which together explain more than 92% of the total variability in the climatic data, were kept as a synthetic variable describing thermal gradients. The species thermal range was calculated as the difference between the average positions of the 10% of the highest and lowest values along this synthetic variable where a given species occurred. We developed multiple stepwise regression models to explore the effects of model accuracy and species traits on the determination of the threshold cutoff.

Cánovas, F., Magliozzi, C., Mestre, F., Palazón, J. & González-Wangüemert, M. (2016) ENiRG: R-GRASS interface for efficiently characterizing the ecological niche of species and predicting habitat suitability. *Ecography,* **39,** 593-598.

## Appendix B. Supplementary figures and tables

### Table B1 Difference in projected changes (relative to baseline) in species ranges among threshold approaches.

|  |  | Range change | Default | MaxKappa | MaxOA | MaxTSS | MiniROCdist | MeanProb | PredPrev=Obs | Sens=Spec |
| --- | --- | --- | --- | --- | --- | --- | --- | --- | --- | --- |
| 2 degree | CT | Total habitat area (km^2^) | a | b | bc | b | bc | c | c | b |
|  |  | Total range change (%) | a | b | b | b | b | b | ab | ab |
|  |  | Habitat gained (%) | a | bc | bc | ab | b | ac | bc | bc |
|  |  | Habitat lost (%) | a | b | b | b | b | b | ab | ab |
|  |  | Eastward shift (m) | a | a | a | a | a | a | a | a |
|  |  | Northward shift (m) | a | bc | bc | ab | b | ac | bc | bc |
|  |  | Uphill shift (m) | a | a | a | a | a | a | a | a |
|  | RT | Total habitat area (km^2^) | a | b | b | b | bc | c | c | c |
|  |  | Total range change (%) | a | b | b | b | b | b | ab | ab |
|  |  | Habitat gained (%) | a | b | b | b | b | a | b | b |
|  |  | Habitat lost (%) | a | bc | bc | bc | bc | b | ac | ac |
|  |  | Eastward shift (m) | a | a | a | a | a | a | a | a |
|  |  | Northward shift (m) | ab | a | a | a | ab | b | ab | ab |
|  |  | Uphill shift (m) | a | a | a | a | a | a | a | a |
| SRE | CT | Total habitat area (km^2^) | a | b | b | b | bc | ac | ab | ab |
|  |  | Total range change (%) | a | a | a | a | a | a | a | a |
|  |  | Habitat gained (%) | a | b | b | b | b | ab | b | b |
|  |  | Habitat lost (%) | a | a | a | a | a | a | a | a |
|  |  | Eastward shift (m) | a | a | a | a | a | a | a | a |
|  |  | Northward shift (m) | a | a | a | a | a | a | a | a |
|  |  | Uphill shift (m) | a | a | a | a | a | a | a | a |
|  | RT | Total habitat area (km^2^) | a | b | bc | bc | ab | a | ac | ac |
|  |  | Total range change (%) | a | a | a | a | a | a | a | a |
|  |  | Habitat gained (%) | a | b | b | b | b | ab | b | b |
|  |  | Habitat lost (%) | a | a | a | a | a | a | a | a |
|  |  | Eastward shift (m) | a | a | a | a | a | a | a | a |
|  |  | Northward shift (m) | a | a | a | a | a | a | a | a |
|  |  | Uphill shift (m) | a | a | a | a | a | a | a | a |

### Table B2 Results of multiple stepwise regression models for the effects of species traits and model accuracy on optimal threshold.

|  | 2 degree | | | | | | |  | SRE | | | | | | |
| --- | --- | --- | --- | --- | --- | --- | --- | --- | --- | --- | --- | --- | --- | --- | --- |
|  | RT | | |  | CT | | |  | RT | | |  | CT | | |
|  | Variable | coefficient | *P*-value |  | Variable | coefficient | *P*-value |  | Variable | coefficient | *P*-value |  | Variable | coefficient | *P*-value |
| MaxKappa | (Constant) | 0.359 | <0.001 |  | (Constant) | 0.354 | <0.001 |  | (Constant) | 0.604 | <0.001 |  | (Constant) | 0.609 | <0.001 |
|  | Latitudinal range | -0.014 | <0.001 |  | Latitudinal range | -0.012 | 0.002 |  | Latitudinal range | -0.025 | <0.001 |  | Latitudinal range | -0.023 | <0.001 |
|  | Marginality | 0.101 | 0.003 |  | Marginality | 0.099 | 0.012 |  |  |  | *R*^2^ = 0.469 |  |  |  | *R*^2^ = 0.364 |
|  |  |  | *R*^2^ = 0.445 |  |  |  | *R*^2^ = 0.325 |  |  |  |  |  |  |  |  |
| MaxOA | (Constant) | 0.359 | <0.001 |  | (Constant) | 0.354 | <0.001 |  | (Constant) | 0.604 | <0.001 |  | (Constant) | 0.610 | <0.001 |
|  | Latitudinal range | -0.014 | <0.001 |  | Latitudinal range | -0.012 | 0.002 |  | Latitudinal range | -0.025 | <0.001 |  | Latitudinal range | -0.023 | <0.001 |
|  | Marginality | 0.101 | 0.003 |  | Marginality | 0.099 | 0.011 |  |  |  | *R*^2^ = 0.469 |  |  |  | *R*^2^ = 0.363 |
|  |  |  | *R*^2^ = 0.446 |  |  |  | *R*^2^ = 0.326 |  |  |  |  |  |  |  |  |
| MaxTSS | (Constant) | 0.359 | <0.001 |  | (Constant) | 0.354 | <0.001 |  | (Constant) | 0.605 | <0.001 |  | (Constant) | 0.609 | <0.001 |
|  | Latitudinal range | -0.014 | <0.001 |  | Latitudinal range | -0.012 | 0.001 |  | Latitudinal range | -0.025 | <0.001 |  | Latitudinal range | -0.023 | <0.001 |
|  | Marginality | 0.101 | 0.003 |  | Marginality | 0.099 | 0.012 |  |  |  | *R*^2^ = 0.470 |  |  |  | *R*^2^ = 0.366 |
|  |  |  | *R*^2^ = 0.446 |  |  |  | *R*^2^ = 0.326 |  |  |  |  |  |  |  |  |
| MinROCdist | (Constant) | 0.363 | <0.001 |  | (Constant) | 0.591 | <0.001 |  | (Constant) | 0.617 | <0.001 |  | (Constant) | 0.631 | <0.001 |
|  | Latitudinal range | -0.014 | <0.001 |  | Latitudinal range | -0.014 | 0.001 |  | Latitudinal range | -0.026 | <0.001 |  | Latitudinal range | -0.027 | <0.001 |
|  | Marginality | 0.103 | 0.004 |  | MXE | -0.197 | 0.013 |  |  |  | *R*^2^ = 0.449 |  |  |  | *R*^2^ = 0.403 |
|  |  |  | *R*^2^ = 0.413 |  |  |  | *R*^2^ = 0.333 |  |  |  |  |  |  |  |  |
| MeanProb | (Constant) | 0.529 | <0.001 |  | (Constant) | -0.157 | <0.001 |  | (Constant) | 0.583 | <0.001 |  | (Constant) | 0.586 | <0.001 |
|  | MXE | -0.264 | <0.001 |  | MXE | 0.881 | <0.001 |  | Latitudinal range | -0.022 | <0.001 |  | Latitudinal range | -0.022 | <0.001 |
|  |  |  | *R*^2^ = 0.294 |  |  |  | *R*^2^ = 0.244 |  |  |  | *R*^2^ = 0.477 |  |  |  | *R*^2^ = 0.447 |
| PredPrev=Obs | (Constant) | 0.338 | <0.001 |  | (Constant) | 0.341 | <0.001 |  | (Constant) | 0.616 | <0.001 |  | (Constant) | 0.586 | <0.001 |
|  | Latitudinal range | -0.012 | 0.001 |  | Latitudinal range | -0.012 | 0.006 |  | Latitudinal range | -0.026 | <0.001 |  | Latitudinal range | -0.022 | <0.001 |
|  | Marginality | 0.122 | 0.001 |  | Marginality | 0.114 | 0.011 |  |  |  | *R*^2^ = 0.431 |  |  |  | *R*^2^ = 0.374 |
|  |  |  | *R*^2^ = 0.389 |  |  |  | *R*^2^ = 0.291 |  |  |  |  |  |  |  |  |
| Sens=Spec | (Constant) | 0.337 | <0.001 |  | (Constant) | 0.341 | <0.001 |  | (Constant) | 0.616 | <0.001 |  | (Constant) | 0.619 | <0.001 |
|  | Latitudinal range | -0.012 | 0.001 |  | Latitudinal range | -0.012 | 0.005 |  | Latitudinal range | -0.026 | <0.001 |  | Latitudinal range | -0.027 | <0.001 |
|  | Marginality | 0.122 | 0.001 |  | Marginality | 0.114 | 0.011 |  |  |  | *R*^2^ = 0.431 |  |  |  | *R*^2^ = 0.375 |
|  |  |  | *R*^2^ = 0.386 |  |  |  | *R*^2^ = 0.292 |  |  |  |  |  |  |  |  |

We also investigated the effects of species traits and model accuracy on the optimal thresholds using multiple stepwise regression models (Table S3). When the 2 degree method was used to select pseudo-absences, the thresholds estimated by the four threshold methods were negatively correlated with latitudinal range and positively correlated with marginality for both CT and RT except for MinROCdist in which the RT threshold negatively correlated with latitudinal range and MXE. For the SRE method, the thresholds determined by the four aforementioned threshold methods increased with decreasing latitudinal range for both CT and RT.

### Table B3 Results of multiple stepwise regression models for the effects of species traits and model accuracy on the difference (standard deviation) in threshold cutoff among eight threshold approaches.

|  | CT |  |  |  | RT |  |  |
| --- | --- | --- | --- | --- | --- | --- | --- |
|  | Variable | Coefficient | *P*-value |  | Variable | coefficient | *P*-value |
| 2 degree | (Constant) | 0.085 | <0.001 |  | (Constant) | 0.072 | <0.001 |
|  | Marginality | -0.035 | 0.001 |  | Latitudinal range | 0.03 | 0.001 |
|  | Latitudinal range | 0.002 | 0.021 |  | Marginality | -0.031 | 0.001 |
|  |  |  | *R*^2^ = 0.316 |  |  |  | *R*^2^ = 0.406 |
| SRE | (Constant) | 0.038 | <0.001 |  | (Constant) | -0.005 | 0.711 |
|  | Latitudinal range | 0.004 | <0.001 |  | Latitudinal range | 0.004 | <0.001 |
|  |  |  | *R*^2^ = 0.274 |  | MXE | 0.050 | 0.013 |
|  |  |  |  |  |  |  | *R*^2^ = 0.459 |

Note: AUC, area under the curve; ROC, receiver operating characteristic curve; SRE, surface range envelop model. CT and RT, classification and regression tree algorithms, respectively.

The multiple stepwise regression models were used to evaluate the effects of species traits and model accuracy on standard deviations of the optimal thresholds of eight threshold approaches (Table 2). Results for both CT and RT showed that the difference in thresholds among eight threshold approaches increased with increasing latitudinal range and decreasing marginality when the 2 degree method was used to select pseudo-absences. When the SRE method was used to select pseudo-absences, the difference in thresholds increased with increasing latitudinal range for CT, while it increased with increasing latitudinal range and MXE for RT.

### Table B4 Results of multiple stepwise regression models for the effects of species traits and model accuracy on spatial correspondence (as judged by the first axis of principal component analysis) in binary predictions produced by eight threshold approaches.

|  |  | CT |  |  |  | RT |  |  |
| --- | --- | --- | --- | --- | --- | --- | --- | --- |
|  |  | Variable | coefficient | *P*-value |  | Variable | coefficient | *P*-value |
| 2 degree | Baseline | (Constant) | 0.702 | <0.001 |  | (Constant) | 0.879 | <0.001 |
|  |  | R^2^ | 0.115 | <0.001 |  | Latitudinal range | -0.005 | <0.001 |
|  |  | Specialization | 0.022 | 0.015 |  | RMSE | -0.157 | 0.010 |
|  |  |  |  | *R*^2^ = 0.312 |  |  |  | *R*^2^ = 0.357 |
|  | Future | (Constant) | 0.726 | <0.001 |  | (Constant) | 0.864 | <0.001 |
|  |  | R^2^ | 0.117 | <0.001 |  | Latitudinal range | -0.005 | 0.01 |
|  |  |  |  | *R*^2^ = 0.229 |  | RMSE | -0.145 | 0.015 |
|  |  |  |  |  |  |  |  | *R*^2^ = 0.332 |
| SRE | Baseline | (Constant) | 0.876 | <0.001 |  | (Constant) | -0.005 | 0.711 |
|  |  | Elevational range | -2.159E-5 | 0.002 |  | Latitudinal range | 0.004 | <0.001 |
|  |  | MXE | -0.068 | 0.019 |  | MXE | 0.050 | 0.013 |
|  |  |  |  | *R*^2^ = 0.244 |  |  |  | *R*^2^ = 0.459 |
|  | Future | (Constant) | 0.908 | <0.001 |  | (Constant) | 0.908 | <0.001 |
|  |  | Longitudinal range | -0.003 | <0.001 |  | Latitudinal range | -0.004 | <0.001 |
|  |  | RMSE | -0.223 | 0.005 |  | RMSE | -0.237 | 0.003 |
|  |  |  |  | *R*^2^ = 0.289 |  |  |  | *R*^2^ =0.395 |

Note: MAE, mean absolute prediction error; RSME, root mean square error; SRE, surface range envelop model.

Multiple stepwise regression models were used to evaluate the effects of species traits and model accuracy on map correspondence. Results from these analyses showed that when the 2 degree method was used to select pseudo-absences, map correspondence was negatively related to latitudinal range and RMSE for RT, while for CT map correspondence was positively related to R^2^ and specilization under current climate and to R^2^ under future climate. When the SRE method was used to select pseudo-absences, for both CT and RT, map correspondence was always negatively related to environmental ranges (elevational, longitudinal, or latitudinal range) and model accuracy measures (MXE, or RMSE) except for RT under current climate it was always positively correlated to latitudinal range and MXE.

### Fig. B1 Differences in threshold and model accuracy among threshold approaches.

### Fig. B2 Frequency of spatial correspondence (as judged by McNemar test) for pairwise among threshold approaches when data were pooled for all species. Data are presented as mean ± SD.

McNemar tests indicated the four aforementioned threshold approaches (MaxKappa, MaxOA, MaxTSS, and MinROCdist) most often yielded a binary map without pairwise significant difference; and the pairwise significant difference most frequently occurred between other approaches and the four threshold approaches or the default 0.5 approaches; intermediate frequency of pairwise significant difference often occurred among the other approaches under current and future climates.

### Fig. B3 Pearson’s correlation for pairwise analysis among model performance measures.

***. Correlation is significant at 0.001 level.

**. Correlation is significant at 0.01 level.

*. Correlation is significant at 0.05 level.
